# Peripartum Cardiomyopathy Presenting as Bradycardia

**DOI:** 10.1155/2017/3670520

**Published:** 2017-02-01

**Authors:** Elisabeth Codsi, Carl H. Rose, Marysia S. Tweet, Sharonne N. Hayes, Patricia J. M. Best, Lori A. Blauwet

**Affiliations:** ^1^Division of Obstetrics and Gynecology, Mayo Clinic, 200 First Street SW, Rochester, MN 55905, USA; ^2^Division of Cardiovascular Diseases, Mayo Clinic, Rochester, MN 55905, USA

## Abstract

Peripartum cardiomyopathy (PPCM) is a disease that typically affects young otherwise healthy women. As PPCM is associated with significant mortality, timely diagnosis is necessary to ensure appropriate care. To our knowledge, this represents the first reported case of PPCM presenting as symptomatic bradycardia. We describe the patient's clinical presentation and relevant findings and review the potential etiology and ramifications of bradycardia in patients with PPCM.

## 1. Introduction

Peripartum cardiomyopathy (PPCM) is a disease affecting young otherwise healthy women near the end of pregnancy or in the early postpartum months [1]. Although incidence is low, patients with PPCM can experience rapid deterioration leading to heart failure, arrhythmias, and even death [1–3]. Because the presenting symptoms are typically nonspecific, clinicians must have a high index of suspicion to ensure a timely diagnosis and proper management. Here we report the first described case of PPCM presenting as symptomatic bradycardia.

## 2. Case Report

A 28-year-old gravida 1 para 1 presented to her local emergency department (ED) on postpartum day 5 for chest heaviness and a “slow heartbeat.” The patient had no significant past medical history, and there was no known family history of cardiac disease or genetic syndromes. There were no complications during pregnancy. The patient delivered a healthy female infant via vacuum-assisted vaginal delivery at 39 2/7 weeks and was discharged home on postpartum day 2. There was no record of intrapartum or postpartum bradycardia during that hospital admission. Initial assessment in the ED revealed a heart rate of 30–40 beats per minute (bpm) and blood pressure (BP) 158/77 mmHg. Atropine 0.5 mg IV was given, causing the patient's heart rate to increase to 80 bpm and systolic blood pressure to rise to 170–180 mmHg. Due to concern for postpartum preeclampsia, labetalol 20 mg IV was administered and a magnesium sulfate infusion was initiated. Computed tomography (CT) of the head showed no acute intracranial event. CT angiography of the chest was negative for pulmonary embolism. Due to concerns regarding the patient's cardiac status, the patient was transferred to our medical center.

Upon arrival to our center, the patient was alert and oriented. Oxygen saturation was normal. Mean arterial pressure remained below 50 mmHg and heart rate ranged between 40 and 60 bpm. A dopamine infusion was initiated and the patient's blood pressure improved. She complained of a worsening headache but no lightheadedness, shortness of breath, or chest pain. Physical exam, including cardiac exam, was unremarkable. Routine laboratory tests were normal. Preeclampsia work-up was negative. Troponin T was 0.01 ng/mL (normal < 0.01 ng/mL) and NT-proBNP was 324 pg/mL (normal < 124 pg/mL). Electrocardiogram showed sinus bradycardia but was otherwise unremarkable. Transthoracic echocardiogram (TTE) revealed a left ventricular ejection fraction (LVEF) of 35%, numerous wall motion abnormalities and an estimated right ventricular systolic pressure (RVSP) of 46 mmHg. These findings suggested possible ischemia or infarction due to multivessel spontaneous coronary artery dissection (SCAD) or PPCM. Repeat troponin T was elevated at 0.03 ng/mL. Aspirin 325 mg was administered. Coronary angiogram showed a 20% lesion in the mid left anterior descending artery but no SCAD. Optical coherence tomography was also negative for SCAD. Right heart catheterization confirmed the RVSP of 48 mmHg.

Dopamine infusion was successfully weaned the following day. The patient's only complaint was dyspnea on exertion. Enalapril was initiated. Cardiac MRI performed on hospital day 3 confirmed left ventricular systolic dysfunction with LVEF of 50% and mild basal hypokinesis but no myocardial delayed enhancement to suggest edema, fibrosis or infarction. The patient was discharged home on hospital day four. Given the constellation of symptoms and diagnostic test results, the final diagnosis was PPCM.

One month after discharge, TTE showed completely normal cardiac structure and function with an LVEF of 63%. The patient reported persistent intermittent bradycardia to 40 bpm mostly during periods of anxiety and in the evenings, without associated dyspnea or chest pain. On 24-hour Holter monitor, her heart rhythm was sinus with occasional sinus arrhythmia and heart rate ranging between 41 and 130 bpm (average, 61 bpm). During a treadmill exercise stress test, the patient achieved 13.0 METS and 113% of predicted functional capacity. ECG showed no signs of ischemia. Heart rate was 89 bpm at baseline, increasing to 173 bpm at peak exercise. Blood pressure was 102/78 mmHg at baseline, increasing to 160/72 mmHg at peak exercise. Enalapril was continued to complete a minimum of 6 months of treatment.

## 3. Discussion

Peripartum cardiomyopathy is defined as cardiac failure occurring in the last month of pregnancy or within 5 months of delivery in the absence of an identifiable cause of heart failure. Additional diagnostic criteria include the absence of heart failure prior to the last month of pregnancy and the evidence of left ventricular systolic dysfunction (depressed shortening fraction or ejection fraction) [1]. In the United States, the incidence of PPCM is 1 in 2,500 to 4,000 live births [3]. Symptoms of PPCM are nonspecific and patients most commonly complain of dyspnea and fatigue. Other presenting symptoms include cough, orthopnea, and pedal edema [1–3]. Because some of these symptoms resemble those of normal pregnancy, diagnosis of PPCM may be delayed. Physical signs are also nonspecific but most commonly include tachycardia, elevated jugular venous pressure, displaced apical impulse, a third heart sound, and a mitral regurgitation murmur [1].

To our knowledge, this is the first reported case of PPCM presenting as symptomatic bradycardia. Typically, the decreased cardiac output observed in PPCM causes an activation of the sympathetic system and a decrease in parasympathetic tone; as a result, tachycardia usually occurs. Although no obvious cause for our patient's bradycardia was identified, our hypothesis is that the bradycardia may have been caused by very high parasympathetic tone. According to some authors, postpartum bradycardia in the absence of PPCM could possibly be genetically related. In a study by Nof et al., 20% of patients presenting with postpartum bradycardia had a mutation of the hyperpolarization-activated nucleotide-gated channel-HCN4, important in the depolarization of the sinus node cells [4]. Other potential causes of bradycardia such as acute myocardial infarction, sick sinus syndrome, infectious causes, exaggerated vagal activity and medications were excluded in this case. Interestingly, the patient continued to experience symptomatic bradycardia more than 2 months following initial presentation, especially at times of increased anxiety.

Reporting this case is of great significance to obstetrician-gynecologists and cardiologists in order to improve our knowledge of the possible presentations of PPCM. Although the incidence of PPCM is low, patients can experience rapid deterioration leading to heart failure, arrhythmias, and even death. The reported mortality rates of PPCM vary significantly between studies, but the range typically varies between 9% and 60% [5–11]. In a population-based study, Mielniczuk et al. reported a much lower mortality rate of 2.05%, attributing this improved prognosis to earlier diagnosis and contemporary management of heart failure [7]. This highlights the importance of a timely and systematic approach, including ECG, TTE, cardiac biomarkers, and possible coronary angiography in cases of bradycardia and decreased left ventricular systolic function in the postpartum state in order to decrease maternal morbidity and mortality.
